# Is the Sun a Magnet?

**Published:** 1892-01

**Authors:** 


### IS THE SUN A MAGNET?

About seventy years ago, Oersted of Copenhagen, found that when a galvanic battery was passed along a wire parallel to a magnetic needle the latter tended to deflect so as to form right angles with the direction of the wire. Tbis discovery formed the foundation of the art of electric telegraphy, and also the ground for a tenable theory in regard to the earth's magnetism. It was argued that the solar rays falling upon the earth carry with them electric force which passes around the earth's surface in a direction parallel with the equator, as our planet turns on its axis daily, that this is the force which causes the magnetic needle to point approximately north and south. This idea has not been materially improved upon in the last half century, though in the course of that time several persons have laid claim to the discovery that the sun is a huge magnet, and found fault with the world for not hailing them as benefactors of the race. Scientific observations of the sun have, however, furnished a great deal of material for sustaining the theory, and explaining some of the phenomena of changes otherwise inexplicable. It has been found that unusual disturbances on the solar surface, whether in the shape of big black spots which are depressions, or eruptive prominences, are followed by deflections of the needle, making what are known as magnetic storms, and that there is at least some connection between them and displays of the aurora borealis.

Some two years ago, Professor Bigelow of Washington undertook a series of investigations into the direction of the lines of force in the solar corona, that brilliant entourage of the sun which is witnessed only during a total solar eclipse. The results of his measurements and mathematical analysis tend to prove that the lines referred to are identical in direction with those in the field of a terrestrial magnet, the rotation of the earth corresponding to the movement in the electricity-inducing dynamo. The parallel is also found to account closely for the well known daily oscillation of the needle in the absence of magnetic storms, the effect varying at any particular spot on the surface as it approaches the sun during the morning hours and then recedes from him in the afternoon. If the earth were stationary the radiant force would be felt immedietely, but owing to its rotation there is a lag of about 23 degrees of arc or an hour and a half of time. On this point observation and the mathematics agree, and it is found that along the resulting curve in the lines of solar force the light and heat pass outward from the sun while the magnetic force is directed inward.

An important theoretical deduction from these comparisons is that if the sun acts magnetically upon the earth through the medium of interstellar ether the earth reacts upon the moon in a similar manner, and by this will possibly be explained certain movements of the lunar nodes which are not satisfactorily accounted for by the theory of gravitation, as well as part of the perihelion motion in the orbit of the planet Mercury. Still another curious point has been brought out, and this settled instead of being simply raised for future solution. It is the rate of rotation of the sun near his poles, which has long been known to be slower than that near the equator. The coronal pole being found to be about 4½ degrees away from the poles of rotation, it has been found that the former rotates once in 27 days, 9 hours, 52 minutes, and 52 seconds.

The results of this study are accepted us valuable by several leading astronomers in this country. An effort will be made to extend the observations, and in particular to examine the relations between magnetism and the weather, for which purpose improved magnetic charts will be supplied to navigators and arrangements made for systematic observations at land observatories. If the theory be substantiated it may result in a wide extention of human knowledge of the relations of worlds to each other, and in a better understanding of "the Newtonian Potential Function in the case of repulsion."

